# Intelligence prediction of microfluidically prepared nanoparticles

**DOI:** 10.1038/s41598-025-21471-y

**Published:** 2025-10-27

**Authors:** Nima Hanari, Sara Mihandoost, Sima Rezvantalab

**Affiliations:** 1 Electrical Engineering Department, Urmia University of Technology, Urmia, 57166‑419 Iran; 2 Chemical Engineering Department, Urmia University of Technology, Urmia, 57166‑419 Iran

**Keywords:** Data mining, Machine learning, Drug loading, Encapsulation efficiency, Nanomedicine, Chemical engineering

## Abstract

Developing poly(lactic-co-glycolic) acid (PLGA) nanoparticles with optimized drug encapsulation and loading is crucial for effective drug delivery. However, controlling the physicochemical properties of these nanoparticles remains challenging. In this study, we compiled a dataset of over 300 PLGA nanoparticle formulations from the literature, including 25 key features related to their preparation on microfluidic platforms. We applied various machine learning algorithms to predict encapsulation efficiency (EE) and drug loading (DL). The random forest model showed the best performance, achieving R² values of 0.93 and 0.96 for DL and EE predictions, respectively. Although EE and DL had minimal impact on each other’s prediction, they provide distinct insights into nanoparticle formulation. Our results demonstrate that machine learning can effectively guide the design of drug delivery systems with desired properties, accelerating their development.

**Supplementary Information:**

The online version contains supplementary material available at 10.1038/s41598-025-21471-y.

## Introduction

Over the past few years, scientists have focused their attention on the development of drug delivery systems (DDSs) with controlled sizes, payloads, and release profiles^1^. In addition to controlling drug concentrations, such DDSs can also reduce harmful side effects associated with the formulation and lead to a reduction in off-target delivery^2^. The amount of DL and EE are key performance indicators in the preparation of DDSs and significantly influence the effectiveness of treatment. Other important factors that influence the overall performance of DDSs include drug release kinetics^3^, stability^4^, biocompatibility^5^, and targeting efficiency^6^. DL refers to the weight of the drug per mass of the nanomedicine, whereas EE refers to the weight of the drug encapsulated in the nanomedicine per initial mass of drug used during preparation^7^. Thus, DL and EE are two key quality attributes describing the amount of drug associated with nanoparticles. While DL is primarily influenced by the carrier material’s structure and chemical/physical properties, EE depends more on the drug-loading mechanism, the amount of drug in the feed, and other experimental conditions^8^. A significant challenge in preparing DDSs is the inefficient delivery of drugs due to low DL and EE of drugs, which can lead to limited pharmacokinetics and inefficient biodistribution^9^. These limitations also impact the cost, manufacturing, toxicity, off-target delivery, and side effects of DDSs^10^. To address these issues, researchers have identified key criteria for improving the delivery efficiency of anti-tumor agents, including material properties, size, shape, zeta potential, active-targeting strategies, and cancer type.

Several strategies have been explored to overcome these challenges^11,12^. For instance, combining physical encapsulation with chemical conjugation has been used to prepare NPs with high DL capacity^13^. However, physicochemical limitations restrict the applicability of this approach for many drugs^14^. Remote loading, a method used for loading doxorubicin into liposomal formulations, is another strategy, but its use is limited by chemical constraints for weak acid or base drugs^15^. For enhancing DL and EE, chemical modifications and surface engineering of drug carriers have also been investigated, including acetylation, glycosylation, amino acid modification, and peptide modification^16^. Microfluidic systems are one of the most successful approaches for controlling NP size and other characteristics^17^. Microfluidic systems have emerged as a promising approach for controlling NP size and characteristics, offering advantages over traditional methods in achieving uniform sizes and narrow size distributions^18^. By precisely controlling the streams, heat and mass transfers are regulated, which are critical for the formation of microparticles and NPs^19^. However, optimizing microfluidic based formulations involves multiple factors, such as flow rate, solvent composition, and interfacial properties, which can vary significantly depending on the type of formulation (e.g., liposomes, polymeric NPs, or lipid NPs)^20,21^. For instance, liposome production via microfluidics can be relatively straightforward with consistent chip designs and controlled parameters, such as lipid composition, hydration time, and flow conditions, enabling reproducible fabrication of liposomes with desired encapsulation efficiency and particle size^22,23^. Microfluidics offers significant advantages in controlling the quality and scalability of formulations which are critical for therapeutic efficacy and regulatory compliance, making it a powerful tool for the production of therapeutics^24,25^. Nevertheless, for more complex formulations or novel systems, identifying an efficient microfluidic synthesis may still rely on empirical approaches and require iterative optimization, which can be time-consuming and resource-intensive. Still, identifying an efficient microfluidic synthesis for a given formulation still relies heavily on empirical approaches and require many trials and errors, which result in time-consuming and expensive experiments. The hierarchy of variables is crucial for preparing DDSs with high DL and EE. How might microfluidic operation conditions or intrinsic material characteristics affect microfluidic operation? In another word, a small change in microfluidic operation conditions, such as flow rate, and channel geometry, as well as intrinsic material characteristics, such as viscosity, interfacial tension, and chemical composition, can significantly shift the outcomes of microfluidic processes. For example, in a very recent study, we considered a wide range of variables including microfluidic operation features (flow rate, and channel geometry), intrinsic material features (e.g. polymer or solvent types), and solution features that can play a substantial role in determining the outcomes of microfluidic processes^26^. We demonstrated that, among a broad range of influencing factors (more than twenty variables) collected from the reported literature, the synthesis method, polyvinyl alcohol (PVA) concentration, and lactide-to-glycolide (LA/GA) ratio of PLGA copolymers are key factors critically influencing NP size. Furthermore, due to time and cost constraints, experimental studies often focus on a limited number of variables to understand how their changes impact the response. In microfluidic settings, numerous variables influence size, DL, and EE, which exhibit complex, nonlinear relationships. Unlike traditional methods such as Design of Experiments (DOE), Machine Learning (ML) can handle and compare a larger number of variables simultaneously, providing a more comprehensive understanding of the factors influencing these outcomes. ML offers a distinct advantage over DOE, especially in scenarios where it is difficult to assess the interplay of multiple variables at once, making it particularly valuable for process optimization in formulation sciences.

Artificial intelligence (AI) and ML are becoming increasingly popular in recent years in order to better understand the conditions and nonlinearities between variables in microfluidics^27^. One of the most studied problems in microfluidics is controlling the size of microparticles or droplets^28–30^. For example, Lashkaripour et al.^30,31^ predicted droplet size using microfluidic geometry and flow conditions. Additionally, fluid characteristics are considered as an effective feature in another follow-up paper^29^ to cover a wide range of variables in the synthesis process on microfluidic platforms as well. Damiati et al.^32^ determined the DL and EE of PLGA-based microparticles containing indomethacin as well as their optimum size and therapeutic loading for microfluidic formulations. Although the training and testing datasets contained few data due to experimental limitations, researchers identified the PLGA concentration and flow rates of both aqueous and organic flows as important parameters in determining PLGA microparticle physiochemical characteristics.

Recently, a number of papers have examined ML models for prediction and/or optimization of EE and DL. For example, encapsulation of glucose oxidase (GOx) and horseradish peroxidase (HRP) in Zn(eIM)2 (eIM = 2-ethylimidazolate) has been optimized through a number of machine learning models, including gradient boosting (GB), support vector machines (SVMs), neural networks (NNs), and RF^33^. It was found that RF provided the best robustness and reliability of prediction among all the models. The authors found out that the synthesis and the optimal composition of biocomposites depend on the enzyme and the overall performance index was enhanced after optimization. The EE of curcumin-loaded liposomes and doxycycline-loaded niosomes was optimized using Least-squares boosting and deep neural network models, respectively^34,35^. As well, the EE of doxorubicin and docetaxel loaded PLGA NPs is predicted using Gaussian Process models with different molecular weight, lactic/glycolic ratio (LA/GA), PLGA: drug ratios, and drug logP parameters^36^. Similarly, the DL of various DDSs has been studied using ML models. A dataset of metal-organic frameworks (MOFs) as carriers and drugs was curated and modeled using multiple machine learning algorithms. According to the results, the metal atoms significantly influenced the drug loading capacity and cytotoxicity of DDSs^37^.

This study uses ML-based models to plot a roadmap for future researchers interested in synthesizing drug-loaded PLGA nanoparticles on microfluidic platforms. Extensive data mining was performed to collect DL and EE parameters of reported PLGA NPs, followed by mathematical analysis to evaluate the impact of each input feature. Lastly, ML ensembles were trained on the screened datasets in order to predict DLs and EEs.

## Materials and methods

### Data mining

We have collected a dataset containing 25 features that may potentially influence the characteristics of the final PLGA-based NPs, as described in the Introduction section. A summary of the features that have been considered in the dataset can be found in Table 1. The dataset was curated through a systematic search of relevant keywords in Web of Science and Google Scholar, ensuring that all references were valid and reliable. Key variables included in the dataset are the molecular weight (Mw) of all polymers (averaged from the reported ranges in the literature), the type and concentration of drugs, the type and concentration of solvents, and the type and concentration of surfactants. Additionally, properties of the microfluidic processes, such as synthesis methods, chip types, channel diameters, and flowrates, were extracted from related publications. To ensure compatibility with machine learning models, we assigned codes to various chips, solvents, and drugs, as reported in the literature. Moreover, in some studies^38^, researchers have utilized two different PLGA polymers (with varying molecular weights), drugs (or therapeutic agents), and solvents. For clarity, we will refer to these as PLGA1 and PLGA2, drug1 and drug2 (In PLGA-based codelivery systems, researchers sometimes incorporated multiple agents such as therapeutic drugs, diagnostic agents, or adjuvants to achieve synergistic effects), and solvent1 and solvent2. Table S1 provides a summary of the drugs, chips, and solvents documented in the literature and collected during the data mining process.

**Table 1:** Features and targets of the curated dataset.

Features			Targets response
Material intrinsic properties	Solution	Microfluidics operation	
Surfactant type	[Drug1] (mg/ml)	Method	EE.(%)
Solvent1 type	[Drug2] (mg/ml)	Total flow (ml/min)	DL.(%)
Solvent2 type	[PLGA1] (mg/ml)	Aqueous flow (ml/min)	
Drug1 type	[Surfactant] (w/v%)	Organic flow (ml/min)	
Drug2 type	[PVA] (w/v%)	Flow ratio	
PLGA1 Mw (kDa)	PEG %	Chip type	
PLGA2 Mw (kDa)	Solvent2/Solvent1	Channel diameter (µm)	
PVA Mw (kDa)			
PEG Mw (kDa)			
PLGA LA/GA			
Size (nm)			

Any parameter enclosed within square brackets [ ] is designated to represent the concentration of the respective component, with all such parameters expressed in units of concentration.

### Feature reduction

In this study, to identify the optimal set of features for EE and DL prediction, two feature selection algorithms—LASSO (Least Absolute Shrinkage and Selection Operator) and Random Forest (RF)—were employed and compared. Below, we briefly describe each algorithm and the corresponding hyperparameters used in our evaluation.

**LASSO** is an effective technique for feature selection in regression problems^39^. It applies an L1 regularization penalty to the model, which drives the coefficients of less significant features to zero, thereby eliminating them from the model. This results in a simpler, more interpretable model that retains only the most important features for prediction. The intensity of the L1 penalty is determined by the alpha hyperparameter, which is set to 1.0 in our analysis.

**RF** is an ensemble learning technique that can be applied for feature selection by evaluating the significance of each feature in predicting the target variable^40^. RF builds several decision trees during training and combines their outputs to enhance prediction accuracy and reduce overfitting. The importance of each feature is determined by how much it helps to decrease impurity, measured by entropy, across all trees in the forest. Features with higher importance values are deemed more critical for the model.

### Regression algorithm

In this study, three widely used regression models—RF, GB, and SVR—were applied for DL and EE prediction. A brief description of each model, along with their corresponding hyperparameters, is provided below.

**RF** is an ensemble learning technique that constructs several decision trees and combines their predictions to enhance accuracy and minimize overfitting^40^. For regression tasks, RF calculates the average of the individual tree predictions to determine the final output. In this study, we set the hyperparameters as follows: n_estimators to 20, max_depth left at its default value of none (allowing trees to grow until all leaves are pure), min_samples_split to 2, and criterion to mean squared error.

**GB** is an ensemble learning method that builds a sequence of decision trees, where each successive tree addresses the errors made by its predecessor^41^. For regression tasks, GB aggregates the predictions of these trees to enhance accuracy and minimize residual errors. The model improves its predictions by concentrating on the data points that were previously mispredicted. The hyperparameter n_estimators (set to 100) specifies the number of boosting stages, while learning_rate (set to 0.1) controls the step size and influences the impact of each tree on the overall model. Additionally, max_depth (default value), along with min_samples_split (set to 2) and min_samples_leaf (set to 1), regulates the model’s complexity and flexibility.

**SVR** aims to find a function that accurately models the data within a specified margin of tolerance while maintaining model simplicity^42^. It accomplishes this by employing a kernel function to transform the input features into a higher-dimensional space, where it fits a regression function. This method allows SVR to capture complex patterns in the data while reducing the risk of overfitting. In this study, after testing various kernels such as RBF and polynomial, we opted for the kernel = linear option.

### Metrics

This study assesses performance using a comprehensive set of metrics, including Mean Absolute Error (MAE), Root Mean Square Error (RMSE), and R-squared (R^2^).

**MAE** computes the average of the absolute differences between predicted and actual values. The formula is:

1 $$MAE=\frac{{\mathop \sum \nolimits_{{i=1}}^{n} \left| {{y_i} - {{\hat {y}}_i}} \right|}}{n}$$

**RMSE** measures the square root of the average of the squared differences between predicted values (${\hat {y}_i}$) and the actual values (${y_i}$). It is calculated using the formula:

2 $$RMSE=\sqrt {\frac{{\mathop \sum \nolimits_{{i=1}}^{n} {{\left( {{y_i} - {{\hat {y}}_i}} \right)}^2}}}{n}}$$

where *n* is the number of data points, ${y_i}$is the actual value for the *i-th* data point, and ${\hat {y}_i}$is the predicted value.

**R**^**2**^ quantifies the proportion of variance in the dependent variable that is predictable from the independent variables. It is an essential measure for assessing model fit, calculated as:

3 $${R^2}=1 - ~\frac{{\mathop \sum \nolimits_{{i=1}}^{n} {{\left( {{y_i} - {{\hat {y}}_i}} \right)}^2}}}{{\mathop \sum \nolimits_{{i=1}}^{n} {{\left( {{y_i} - \underset{\raise0.3em\hbox{$\smash{\scriptscriptstyle-}$}}{y} } \right)}^2}}}$$

where $$\underset{\raise0.3em\hbox{$\smash{\scriptscriptstyle-}$}}{y}$$ represents the mean of the actual values.

## Results and discussion

### Data preparation

The dataset was split into training and test sets using an 80/20 ratio. A feature selection algorithm was applied to the training data to identify the optimal feature set. Afterward, the training data was normalized, and the selected features and normalization parameters were applied to the test set. The test set, which remained independent throughout the normalization and training processes, was then used to evaluate the performance of the proposed method.

### Feature selection

**Fig. 1:**
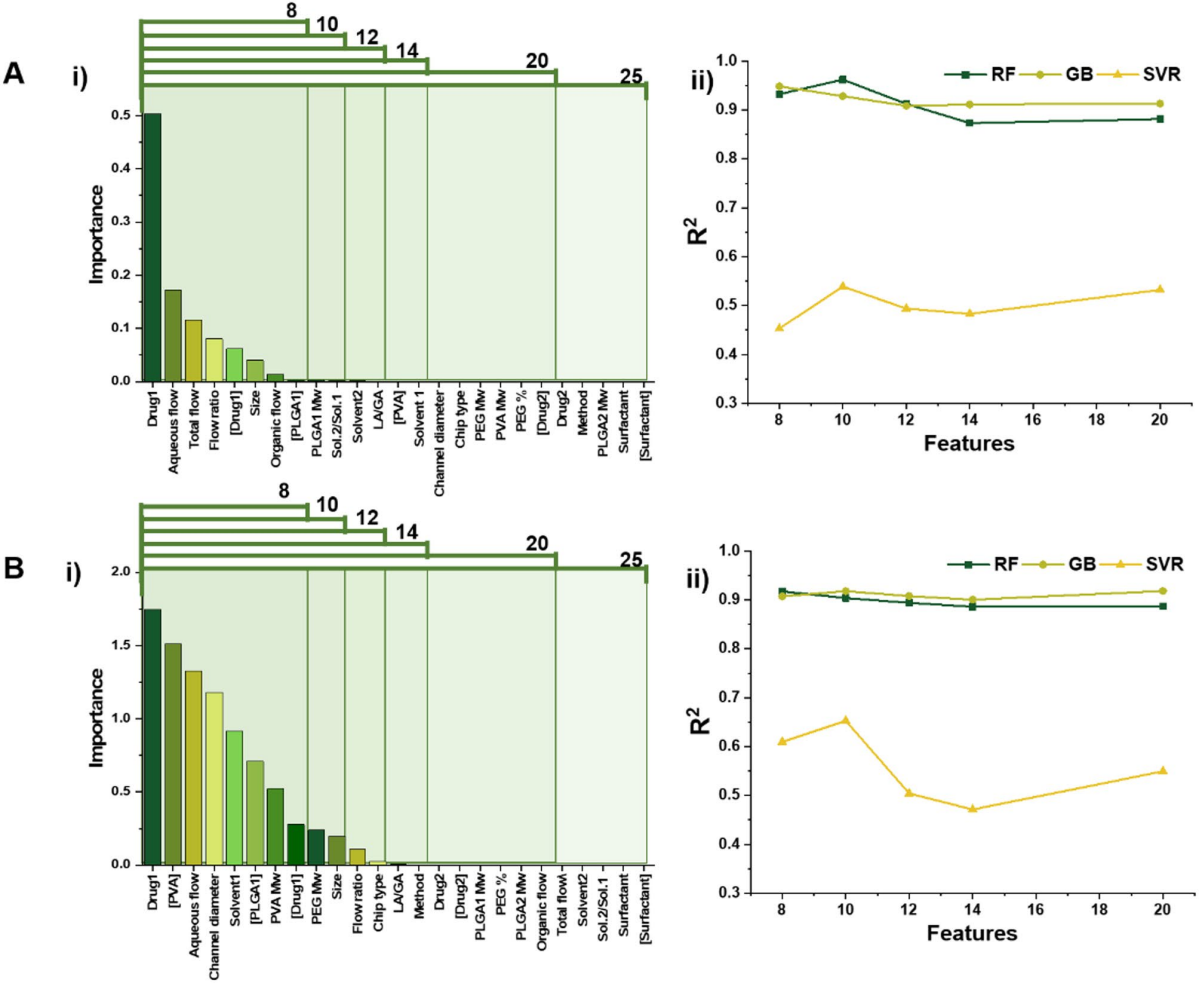
Feature reduction. A-i) Feature importance calculated through RF feature selection. **A-ii)** Feature reduction based on the priority and scrutinizing the impact of each stage reduction on the prediction of DL. As can be seen, among RF regressor, GB, and SVR models with 10 features using GB resulted in high R^2^ scores. Increasing the feature numbers didn’t improve the score for RF model significantly. **B-i**,** ii)** feature reduction based on LASSO model and investigation of different sets of features on the R^2^ score of SVR, GB, and RF models. Similarly, increasing the number of features after 10 has no significant impact on R^2^ score of RF regressor.

After preprocessing, we began by evaluating the effectiveness of the features. Two feature selection methods, RF and LASSO, were used to identify the most important parameters. Figure 1 presents the results of both methods for predicting DL. Each method assigned different importance scores to the features, and we removed those with the lowest absolute importance scores in five stages. We then assessed the performance of the remaining features across three models: SVR, GB, and RF regressors. Specifically, we tested the models using 20, 14, 12, 10, and 8 features to determine whether reducing the number of features had a positive or negative impact on performance. A higher R² score at each stage indicated that the features selected by the RF method provided better predictions. As shown in Fig. 1, the most important feature in both the RF and LASSO models was “drug1,” suggesting that the main drug type, as reported in the literature, plays a significant role in DL values. This result is reasonable, given that the type of drug, along with its properties and interactions with the carrier, significantly influences DL outcomes. Both methods also identified surfactant type and surfactant concentration as the least important factors in determining DL. Moreover, the results highlighted that NP size, aqueous flow rate, and drug1 concentration were important features common to both methods, emphasizing their importance for further investigation. These findings align with previous research, which pointed to NP size as a key factor in DL and drug delivery effectiveness. Additionally, PLGA concentration in the organic solution emerged as another crucial parameter influencing DL.

The organic flow rate is among the selected features that regulates DL in PLGA NPs synthesized via microfluidics, as it directly governs the nanoprecipitation kinetics. Higher flow rates typically lead to faster mixing with the aqueous phase, promoting rapid solvent diffusion and polymer precipitation, which can entrap drug molecules more efficiently before they diffuse out of the forming NPs. Conversely, slower flow rates may result in incomplete drug encapsulation due to prolonged solvent-water interdiffusion, leading to drug leakage. Our analysis identified organic flow rate as a key parameter influencing DL, underscoring its role in controlling supersaturation and nucleation rates during NP formation. Moreover, the solvent ratio (sol.2/sol.1) emerged as another significant factor affecting DL (as in some reports^43^, researchers used binary solvent systems e.g., dimethyl sulfoxide (DMSO) and dichloromethane (DCM) mixtures for the preparations). The relative proportions of solvents modulate polymer solubility, drug partitioning, and precipitation dynamics. For instance, a more volatile solvent (e.g., DCM) paired with a slower-diffusing one (e.g., DMSO) can fine-tune the rate of PLGA deposition, thereby influencing drug entrapment efficiency. Our data revealed that an optimal solvent ratio maximizes DL by balancing rapid NP solidification (to prevent drug loss) with sufficient drug–polymer interaction time.

These findings highlight that while the absolute type of solvent may have a secondary impact on DL compared to other factors (e.g., [drug]), solvent-associated process parameters (such as flow rate and sol.2/sol.1ratios) are pivotal in optimizing drug incorporation. This aligns with prior studies demonstrating that microfluidic mixing conditions and solvent composition dictate NP properties by affecting interfacial turbulence, supersaturation gradients, and final NP morphology. In conclusion, as illustrated in Fig. 1, we chose to proceed with further analysis based on the RF coefficients. The test sets with 8 and 10 features yielded R² scores of 0.932 and 0.962, respectively. Based on these results, we selected 10 features for continued analysis.

**Fig. 2:**
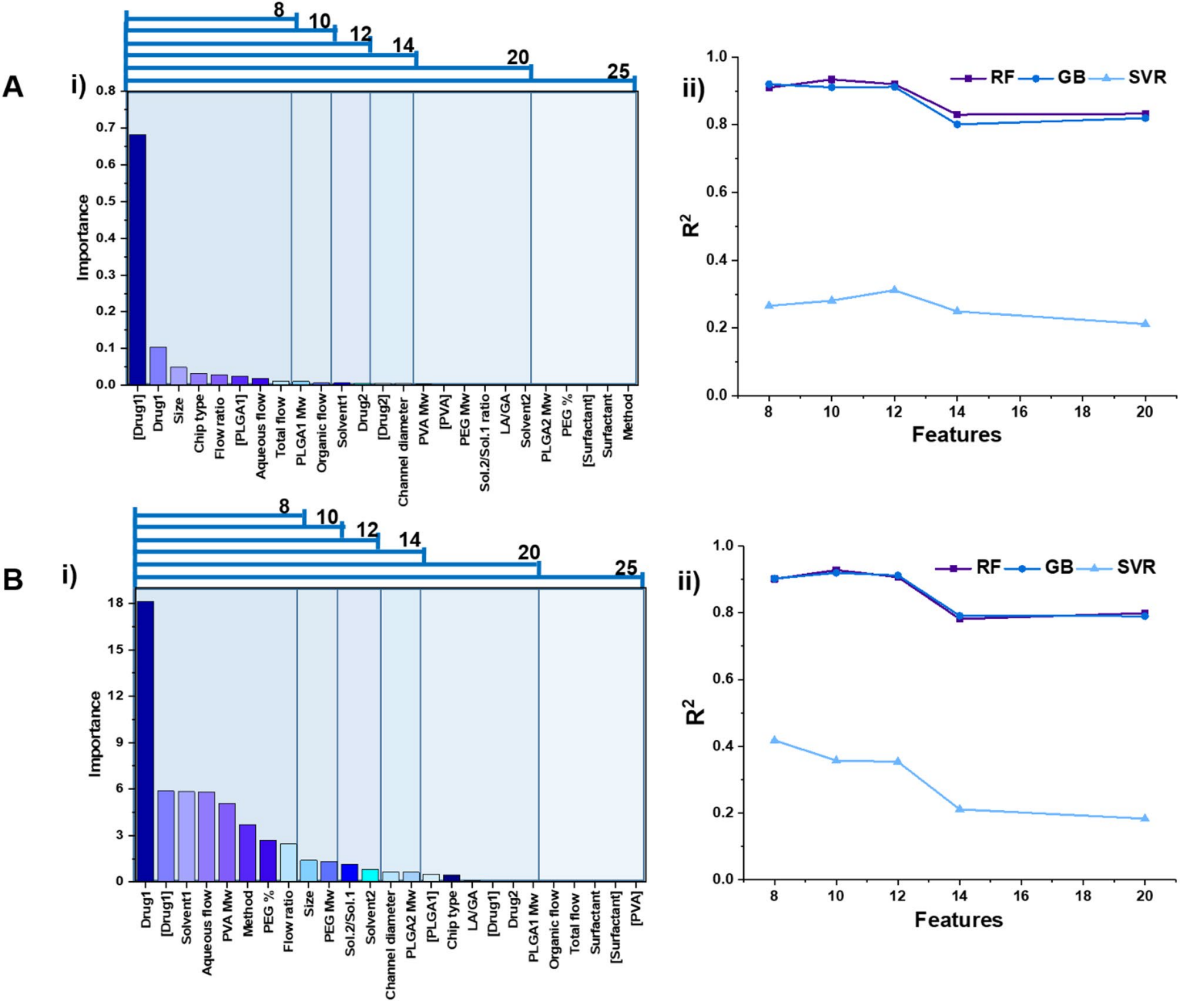
**A-i)** Feature importance calculated through RF feature selection. **A-ii)** Feature reduction based on the priority and scrutinizing the impact of each stage reduction on the prediction of DL. As can be seen, among RF regressor, GB, and SVR models with 10 features using GB resulted in high R^2^ scores. Increasing the feature numbers didn’t improve the score for RF model significantly. **B-i**,** ii)** feature reduction based on LASSO model and investigation of different sets of features on the R^2^ score of SVR, GB, and RF models. Similarly, increasing the number of features after 10 has no significant impact on R^2^ score of RF regressor.

A similar approach was applied to evaluate the influence of feature reduction on the prediction of EE (Fig. 2). Feature reduction was performed using both RF and LASSO, as shown in Fig. 2. Notably, the main drug type and its concentration were identified as the top two most influential features in both methods. Conversely, surfactant type and surfactant concentration ranked among the least influential factors. Key features affecting EE include drug type and concentration, NP size, and chip type that are critical in determining the drug encapsulation mechanism into PLGA.

For instance, chips with laminar flow designs, such as flow-focusing configurations^44^, allow for precise control over mixing and nanoparticle formation, leading to more uniform particle sizes and better DL^45^. In contrast, chips that induce turbulent flow may result in less control over particle size distribution, potentially reducing EE. Additionally, the mixing efficiency of the chip is crucial; designs with rapid mixing, such as herringbone^46^ or chaotic mixers^47^, can enhance the interaction between the drug and PLGA, improving EE^48^. Poor mixing, on the other hand, can lead to uneven drug distribution within the NPs^49^. Moreover, the chip design must ensure efficient mixing of the organic phase (containing polymer and drug) with the aqueous phase to form stable NPs^50^. Operational features such as flow rates, and residence time can influence EE. Since higher flow rates can lead to smaller NPs with a higher surface area, potentially increasing DL but may also reduce EE if the drug is not well incorporated. Lower flow rates allow for better control over NP size and DL^51^.

Various surfactants have been used in both bulk and microfluidic preparation of PLGA NPs, and previous studies have reported differing effects depending on the surfactant type^52–54^. For example, non-ionic surfactants, including PVA^55^, Poloxamers (Pluronic^®^ F68^56^, F127^53^), and Tween 80^57^, are widely used. PVA stabilizes NPs by forming a hydrophilic coating that prevents aggregation^58^, while Poloxamers enhance EE for hydrophobic drugs through micelle-mediated drug incorporation^59^ and improve cellular uptake due to their stealth properties^60^. Tween 80 increases DL for lipophilic compounds by reducing interfacial tension^61^. Ionic surfactants like sodium cholate^62^ and sodium dodecyl sulfate^55,63^ provide strong stabilization but face limitations due to cytotoxicity, restricting their biomedical use. In contrast, amphiphilic surfactants such as lecithin^64^ and DSPE-PEG^65^ offer improved biocompatibility and functionality. For instance, lecithin enables higher DL by forming hybrid lipid-PLGA NPs^66^. Moreover, the surfactant concentration plays a crucial role in determining NPs properties. Insufficient surfactant levels result in unstable emulsions, larger NPs, and lower EE. An optimal surfactant concentration improves NP characteristics, including size, polydispersity, and EE. However, excessive surfactant can lead to micelle formation, competing with NPs for drug loading and altering drug release kinetics^53^.

On the other hand, our results from applying ML methods for feature importance indicate that the surfactant and its concentration have minimal impact on PLGA NP preparation in microfluidic settings. The observation can be attributed to the dominant role of hydrodynamic control in microfluidic systems. In microfluidics, the flow rates (aqueous-to-organic phase ratio, total flow rate) and chip geometry (e.g., mixing design, channel dimensions) primarily dictate the mixing efficiency, diffusion kinetics, and nanoprecipitation dynamics. These parameters directly influence the supersaturation rate of PLGA and the drug, governing nucleation, growth, and the final drug-polymer matrix formation^67^ as the key determinants of DL and EE. Since microfluidics enables precise control over these processes, the influence of surfactants (which primarily act as stabilizers) becomes secondary once the core NP structure is established during rapid nanoprecipitation. Unlike bulk methods such as emulsion evaporation, where surfactants are critical for droplet stabilization and preventing coalescence^68^, microfluidics relies more on hydrodynamic flow confinement to achieve reproducible NP formation with narrow size distributions^49^. As a result, the surfactant’s role shifts from actively controlling particle formation to providing post-synthesis colloidal stability. ML models identified flow rates and chip type as higher-importance features due to their direct impact on shear forces, mixing time, and diffusion-limited assembly. Surfactant properties may have exhibited a saturation effect beyond a minimal concentration required for stabilization. Additional surfactant does not significantly alter DL or EE.

It is worth mentioning that while chip type emerged as a significant factor affecting EE, channel diameter showed minimal impact on both EE and DL. This finding suggests that the functional characteristics of the microfluidic device, particularly those governing mixing dynamics, play a more crucial role than simple geometric dimensions in determining NPs quality. The importance of chip type aligns well with established principles of microfluidic NPs synthesis. Different chip architectures, such as flow-focusing^69^, herringbone^70^, or Tesla mixer designs^48^, create distinct fluid dynamics that directly influence the nanoprecipitation process. Flow-focusing chips generate controlled laminar flow conditions that enable precise size control^71^, while chaotic mixers with herringbone structures or 3D obstructions promote more efficient mixing through induced turbulence^72^. These mixing characteristics appear critical for drug encapsulation, as they determine the interaction time between the drug and polymer phases^73^. Recent studies^48^ have demonstrated that optimized mixer designs can improve EE by up to 20% compared to standard configurations, supporting our model’s identification of chip type as a key parameter. In contrast, the relatively minor importance of channel diameter in our models suggests that its effects may be compensated for by other parameters. While channel diameter theoretically affects flow velocity and shear forces, in practice, these effects can often be replicated by adjusting flow rates in different chip configurations. The curate dataset reflects a limited range of channel diameters (typically 20–2000 μm in most studies), reducing the observable impact of this parameter.

Our model’s findings align well with experimental studies, as they are consistent with previous research results^8^, demonstrating the reliability and validity of our approach. Specifically, this methodology can be utilized to optimize the design of drug delivery systems by identifying critical parameters that influence EE^54,74–76^. For instance, our approach provides a more systematic and data-driven way to prioritize experimental variables compared to traditional trial-and-error methods, potentially reducing development time and costs. Additionally, the identification of drug type and concentration as top features offers a clear direction for further experimental validation and refinement of PLGA-based drug delivery systems. This improvement over standard practices highlights the practical utility of our model in advancing drug formulation research.

It is worth mentioning that our study aimed to prioritize the most influential parameters affecting the properties of PLGA NPs from a comprehensive set of potential features. Our findings may differ from those of other studies, as experimental research often focuses on a narrower range of variables to ensure feasibility and applicability. In contrast, our approach involved an extensive review of the literature, enabling us to compile a broad dataset encompassing numerous features. These features were systematically analyzed and managed using advanced ML methods, providing a more holistic understanding of the factors influencing EE in PLGA NPs.

**Fig. 3:**
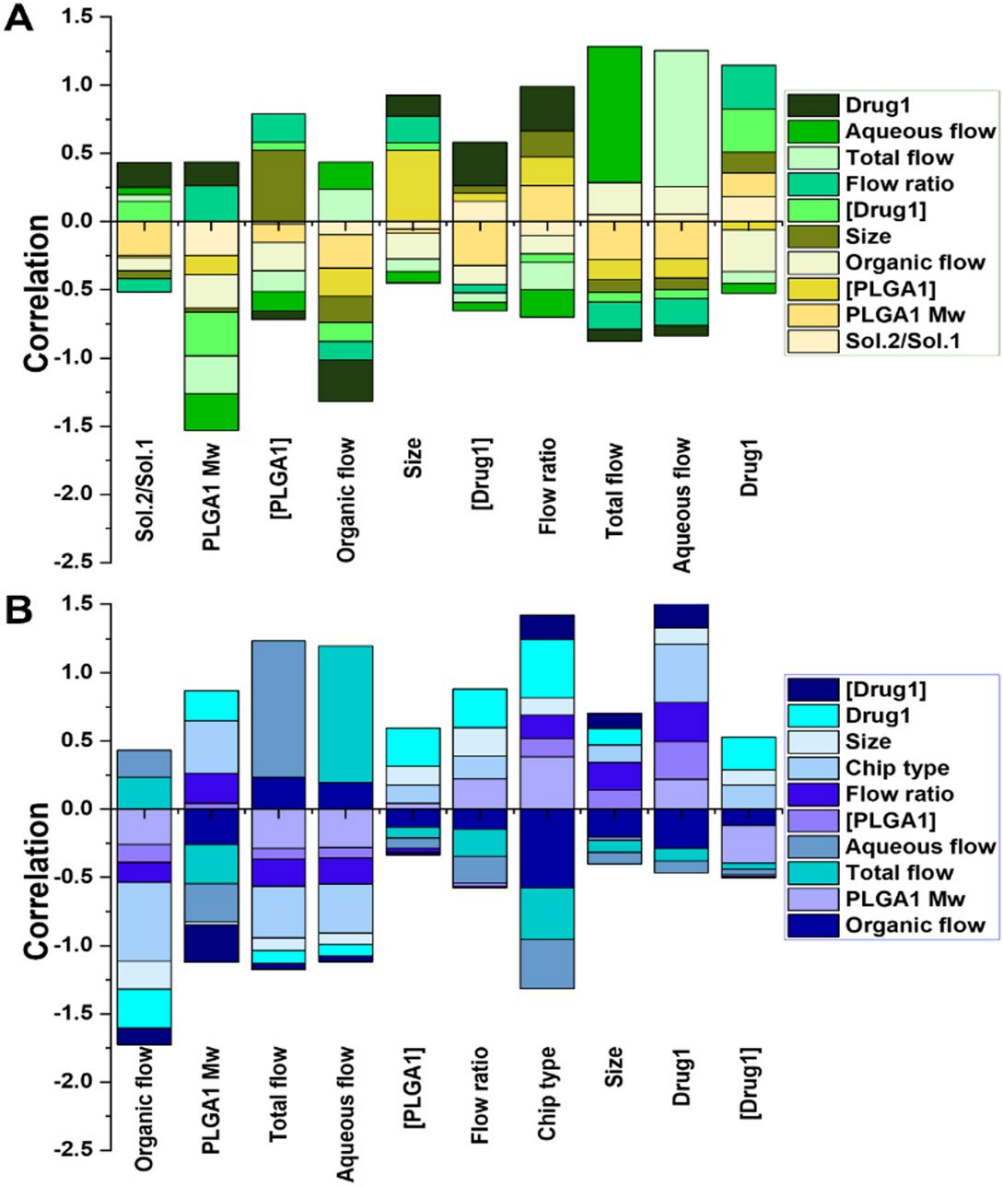
**A**,** B)** Correlation between selected features for modeling of DL and EE, respectively.

Additionally, a pairwise correlation analysis was performed to quantify the correlation between the selected features screened for both DL and EE (Fig. 3). As illustrated in Fig. 3A, the strongest positive correlation is observed between the total flow rate and the aqueous flow rate. This finding is both logical and anticipated, as the total flow rate is the sum of the aqueous and organic flow rates. An interesting correlation between size and [PLGA1] is also observed, indicating that PLGA concentration influences the size of NPs and an increase in the PLGA concentration leads to the formation of bigger NPs. For instance, Xu et al.^43^ demonstrated that higher PLGA concentrations resulted in larger NP sizes. Their findings revealed a significant increase in mean NP volume with rising PLGA concentrations, highlighting a direct correlation between concentration and size. Similarly, another study^77^ observed that increasing PLGA concentrations from 1 mg/mL to 10 mg/mL caused NP sizes to grow from 65 nm to 150 nm. The authors explained this phenomenon by noting that higher concentrations increased the viscosity of the organic phase, which reduced diffusivity and prolonged mixing times, ultimately favoring the formation of larger particles. Furthermore, when considering the correlations between features of EE, the highest positive correlation is seen with total/aqueous flow rates. Drug1 and chip type also demonstrated positive correlations with each other as well as with PLGA1 concentration and Mw.

Readers are encouraged to explore the review paper^78^ on properties and applications of PLGA NPs in nanomedicine, which highlights various barriers and challenges that must be addressed to facilitate clinical translation. It is important to note that DL and EE are just two of the many critical metrics emphasized in current research. By identifying and optimizing the key parameters that influence DL and EE, our goal is to develop NPs with improved drug incorporation and retention properties. This foundational research is a crucial step before advancing to subsequent studies that assess the delivery efficacy of these NPs in biological systems.

### Regression model selection

After selecting the optimal set of features using the RF feature selector, we evaluated the performance of different regression models for predicting DL and EE using the best set of selected features. Table 2 presents the results of applying three regression models SVR, GB, and RF measured in terms of RMSE and R^2^. As shown in Table 2, the RF model outperformed both SVR and GB for predicting DL and EE. Moreover, the RMSE and R^2^ values for both the training and testing sets demonstrate that the models were well-trained, with no signs of overfitting.

**Table 2:** Model performances through metrics for DL and EE predictions, based on 10 features that have been selected through RF classifier.

	Model	RMSE		R^2^	
		Train	Test	Train	Test
DL	RF	0.79	1.01	0.97	0.96
	GB	0.24	1.91	0.99	0.92
	SVR	4.04	4.77	0.35	0.15
EE	RF	4.62	11.07	0.97	0.93
	GB	3.81	11.77	0.98	0.90
	SVR	23.39	24.17	0.23	0.24

To further address the potential concern of overfitting due to the limited sample size (*n* = 300) and feature space, we conducted a 5-fold cross-validation using the reduced 10-feature set obtained through RF selection. The cross-validated RMSE results, along with their statistical significance, are summarized in Table 3. For the DL target, RF showed the lowest cross-validated RMSE (1.36 ± 0.74), followed by GB (1.42 ± 0.61), while SVR had significantly higher RMSE values (4.16 ± 1.53). A similar trend was observed for the EE target, where RF and GB achieved comparable performance (11.39 ± 2.60 for RF vs. 11.35 ± 2.62 for GB) while both significantly outperformed SVR (23.70 ± 4.49).

In addition, to evaluate the robustness and statistical reliability of model predictions across cross-validation folds, paired t-tests and Wilcoxon signed-rank tests were applied for both DL and EE targets, as shown in Table 3. For DL, no significant difference was observed between RF and GB (t-test *p* = 0.99, Wilcoxon *p* = 0.81), but RF significantly outperformed SVR (t-test *p* = 0.01, Wilcoxon *p* = 0.06). For EE, the RF model performed comparably to GB (t-test *p* = 0.81, Wilcoxon *p* = 0.81) but significantly outperformed SVR (t-test *p* = 0.00, Wilcoxon *p* = 0.06). These results support the stability and generalization capability of the selected RF-based models and confirm the effectiveness of cross-validation in controlling for overfitting.

**Table 3:** Model performances through t_test and Wilcoxon signed-rank tests for DL and EE predictions, based on 10 features that have been selected through RF selector.

	Model	5-fold cross-validation RMSE		Model	Statistical test	
		Train Test			t_test p-value	Wilcoxon p-value
DL	RF	1.36 ± 0.74	DL	RF vs.GB	0.99	0.81
	GB	1.42 ± 0.61				
				RF vs.SVR	0.01	0.06
	SVR	4.16 ± 1.53				
EE	RF	11.39 ± 2.60	EE	RF vs.GB	0.81	0.81
	GB	11.35 ± 2.62				
				RF vs.SVR	0.00	0.06
	SVR	23.70 ± 4.49				

**Fig. 4:**
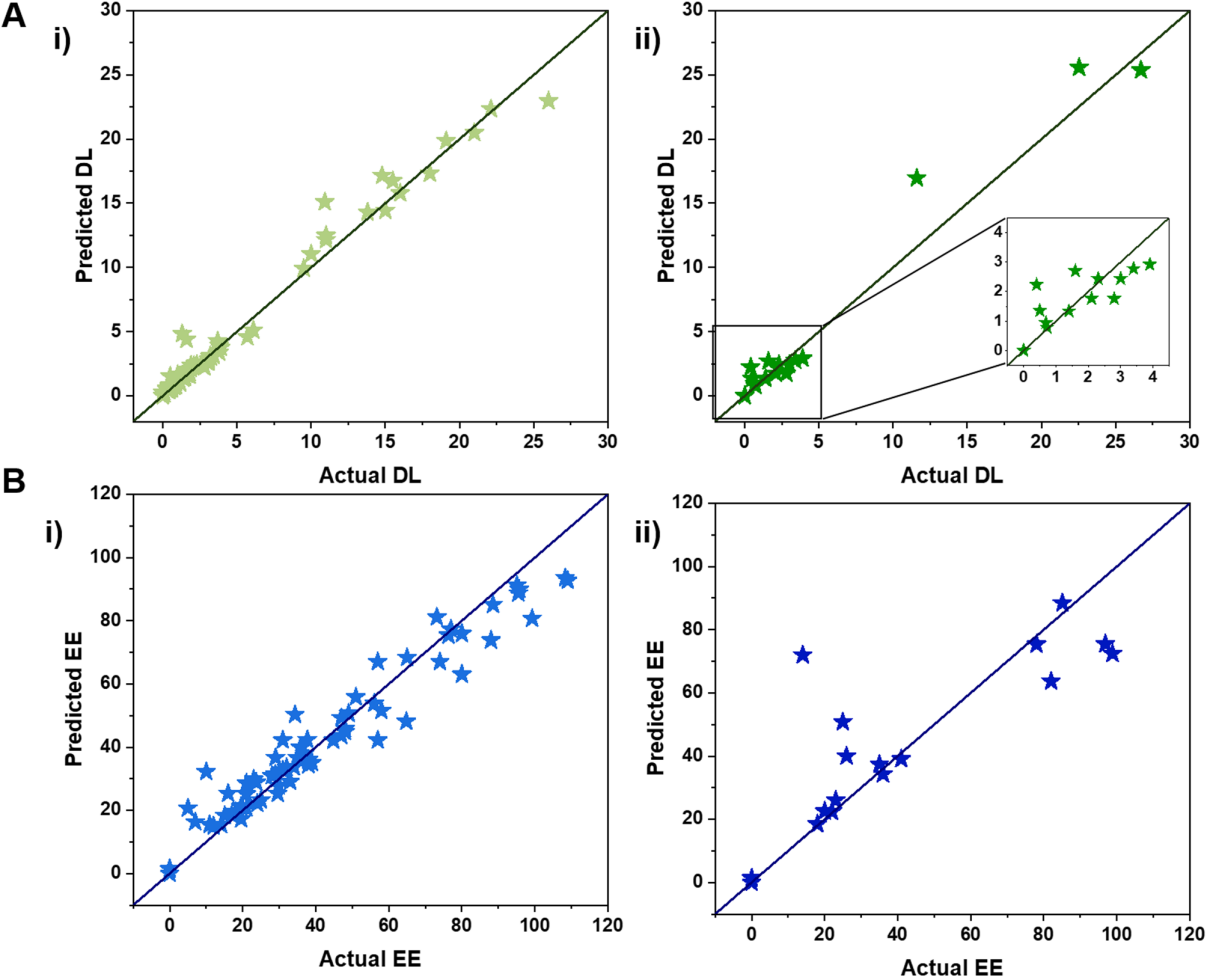
Selected model performances. **A-i**,** ii)** Comparison of the predicted and actual values of DL in the training and testing sections, respectively. **B-i**,** ii)** Comparison of the predicted and actual values of EE in the training and testing sections, respectively.

Additionally, Fig. 4 illustrates the predicted versus actual values for both DL and EE in the training and test sets. Consistent with previous results, the prediction points for DL are closely distributed around the bisector in both the training and test sets, further supporting the model’s lower MAE and higher R^2^ score. However, for EE, the predicted and actual values show slight discrepancies, as indicated by the wider distribution of points beyond the bisector line.

**Fig. 5:**
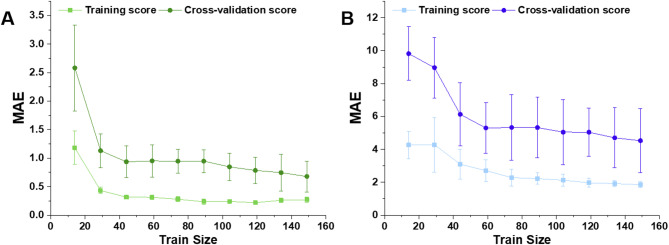
Cross-validation analysis on the model performances. **A**,** B)** Sample size impact on the performance of selected models from previous section for the prediction of DL and EE, respectively. Apparently, with increase in the training dataset set, MAE for both decrease.

Furthermore, to visually validate model generalization, learning curves for both EE and DL predictions were plotted to verify the absence of overfitting. The MAE for the training and validation data was calculated across varying amounts of training data. As shown in Fig. 5, the convergence of the training and validation curves confirms that the models were trained without overfitting.

### Interrelationship between DL and EE: impact analysis

**Fig. 6:**
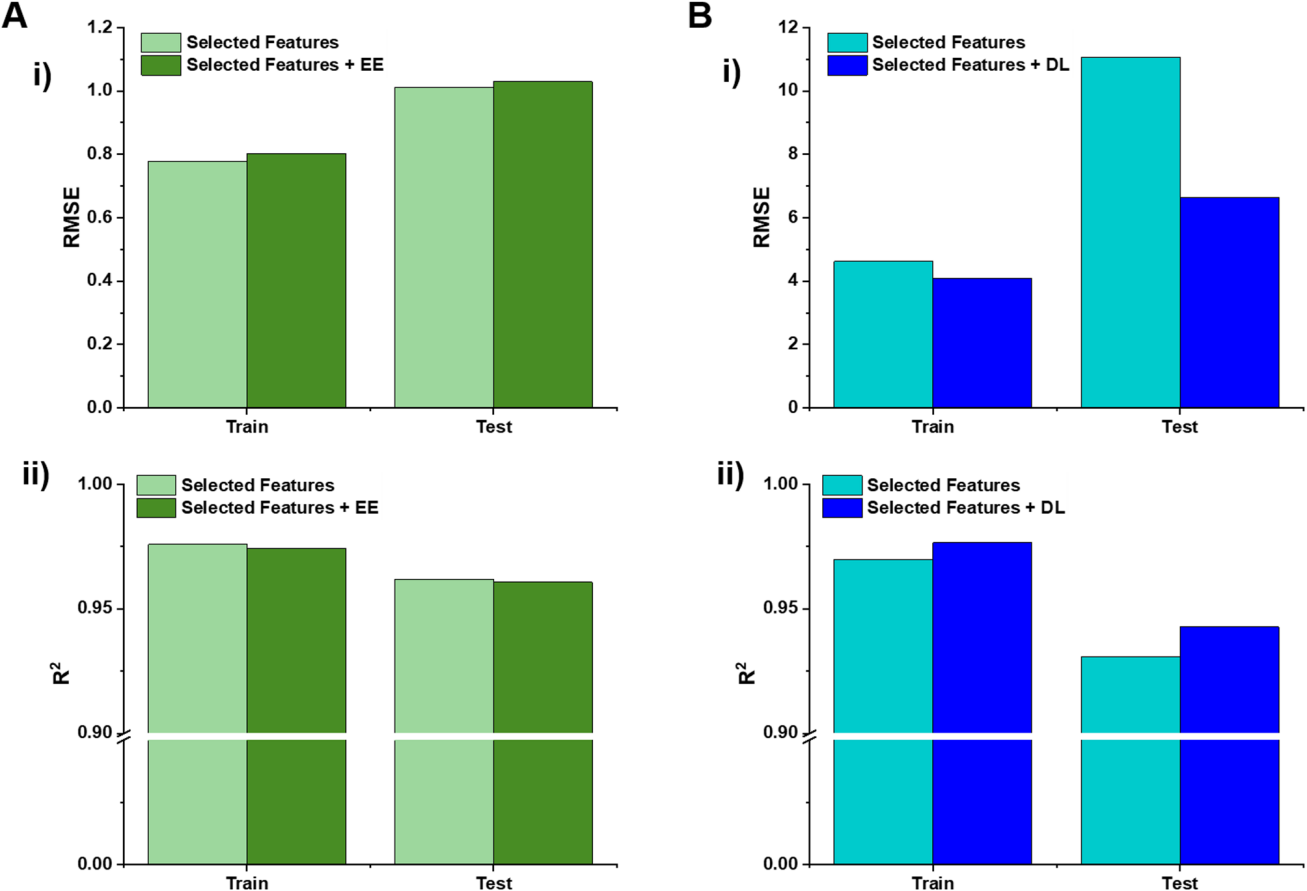
Impact of DL and EE on each other. **A-i**,** ii)** RF model metrics RMSE and R^2^ score with addition of EE as new feature to the selected dataset, respectively. **B-i**,** ii)** Presence of DL as additional feature in the selected dataset on the model metrics including RMSE and R^2^ score, respectively.

To assess whether DL and EE significantly impact one another, we performed additional modeling by incorporating EE as a feature when predicting DL, and vice versa, adding DL as a feature when predicting EE. The results of these analyses are illustrated in Fig. 6.

The first step was to compare the performance of the RF model with the selected features and then with the addition of EE to the reduced feature set for predicting DL. The results show that the RMSE of the model increased slightly, from 0.78 to 0.80 during training and from 1.01 to 1.03 during testing. Meanwhile, the R² scores exhibited negligible changes, decreasing marginally from 0.98 to 0.97 during training and from 0.962 to 0.961 during testing. Based on this small increase in RMSE and minimal drop in R^2^, it can be concluded that EE does not have a significant positive influence on DL prediction.

Next, DL was added as a feature to evaluate its influence on EE prediction. When DL was included, the RMSE of the RF model decreased slightly from 4.62 to 4.08 during training and from 11.07 to 6.62 during testing. Additionally, the R² scores improved slightly, rising from 0.97 to 0.98 in the training phase and from 0.93 to 0.94 in the test phase. While this indicates a small positive impact of DL on EE prediction, the effect is minimal and can be considered negligible.

In line with our previous findings^79^, these results suggest that DL and EE have no significant impact on each other.

### Local explanation

**Fig. 7:**
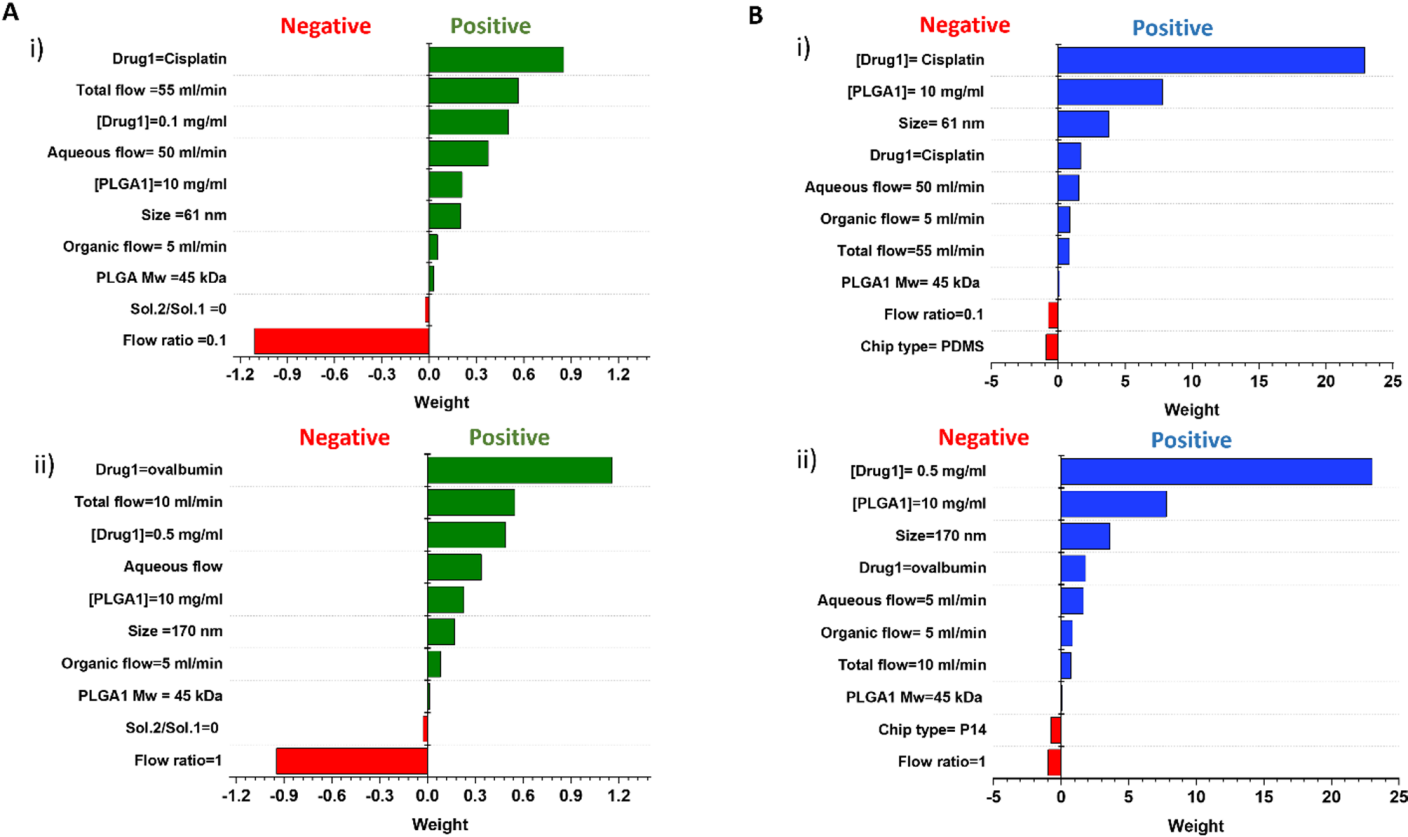
LIME feature importance for randomly selected samples from test set for DL and EE. **A-i**,** ii**) two different from the test set for DL. **B-i**,** ii)** Two different samples from the test set for EE.

Figure 7 presents the explanation of the 10 most important features for two examples from the test set in predicting DL and EE. LIME plots were utilized to highlight the relationship between the RF model’s predictions of DL and EE values and the selected features. In the green and blue sections of the diagrams, the positive impact of features on DL and EE predictions, respectively, is evident, while the red bars indicate the negative impact of features.

For instance, in the case of DL prediction, an increase in the flow ratio leads to a decrease in DL values (see Fig. 7, B-i, ii). This observation can be attributed to the fact that an increased flow ratio corresponds to a higher organic flow containing polymer strands and drugs, which impedes solvent diffusion and component displacement. As a result, the accessibility of drug molecules within the organic solvent diminishes, leading to a reduction in DL values. Additionally, the use of a combination of two solvents can negatively affect DL values for PLGA NPs, as the solvents influence the formation of PLGA NPs differently. This process may result in the loss of drug molecules and, subsequently, a decline in DL values.

A similar trend is observed for EE predictions. An increase in drug and PLGA concentrations enhances EE values, which aligns with experimental expectations. Furthermore, size emerges as a critical factor influencing EE values, as supported by prior research^79^. The results indicate a direct correlation between EE and the size of PLGA NPs, with larger sizes resulting in higher EE values.

In conclusion, the flow ratio has a detrimental effect on both DL and EE. Future researchers synthesizing PLGA NPs using microfluidics should be mindful of this negative impact and strive to maintain lower flow ratios to optimize DL and EE values.

### Study limitations

The present study presents the application of ML methods to more intelligently design PLGA-based NPs as DDSs in microfluidic settings. Apart from the obtained promising results, the data mining method has some limitations due to the quality and quantity of the gathered training data.

As for the quantity of the data, we were able to access about three hundred data points with full descriptive information concerning DL and EE of PLGA-based NPs. However, ML methods rely on a large number of training data, as the larger the number of training data, the better the quality of the prediction.

Generally, quality of the data is described as the impact of various experimental conditions and laboratory equipment as well as a variety of operational skills on the reported and consequently gathered data. Insufficient data and human and equipment errors can therefore be associated with the curated dataset, which can subsequently affect the models. On the other hand, preparing an ideal dataset with a large number of data points can be time-consuming and costly, especially when considering the use of hazardous and expensive materials. Considering this, the data mining methodology from literature is a more appropriate approach for obtaining a deeper understanding of the role played by multiple parameters. As evidence of the validity and applicability of the presented study, a similar approach was taken when considering the (cyto)toxicity of the NPs^80–83^, discovery and synthesis of nanomaterials^84,85^, nanomedicine^86,87^.

Future research directions could focus on incorporating additional data sources, exploring different ML architectures, or validating the model with experimental data. It is also essential to document and standardize the software configuration used in this study to enhance reproducibility and facilitate further advancements in this field.

### Comparison with DOE and advanced ML methods

Traditional DOE methods—such as factorial design and response surface methodology—have long been utilized for optimizing nanoparticle formulations. However, these approaches often require a high number of experiments and struggle to model complex, nonlinear relationships among multiple variables. In contrast, the ML based models employed in this study achieved high predictive accuracy using only 300 data points and a reduced set of 10 key features, significantly minimizing the need for extensive experimental trials.

Previous studies, such as Sedighi et al.^23^, have demonstrated the effectiveness of combining DOE with microfluidic systems for nanoparticle optimization. Nevertheless, our findings highlight that ML methods—especially ensemble-based models—offer a more efficient alternative, capable of capturing intricate interactions among variables. With R² scores exceeding 0.93, our models outperformed what could typically be achieved through conventional DOE approaches without requiring large-scale experimental campaigns.

Although advanced techniques such as deep neural networks (DNNs) and hybrid frameworks (e.g., DOE-ML integrations or generative adversarial networks) have been explored in the literature (see section “Introduction”), we focused on interpretable models in this study, given the constraints of data volume. Future research may benefit from investigating such hybrid strategies to combine the strengths of both experimental design and deep learning, potentially improving prediction accuracy and expanding model generalizability.

## Conclusion

The production of drug-loaded nanoparticles (NPs) with high DL and EE is essential for the success of drug delivery systems (DDSs). Achieving these critical quality attributes (CQAs) requires the careful balancing of numerous formulation and process variables—traditionally optimized through trial-and-error or DOE frameworks, both of which can be time-consuming and resource-intensive. In this study, we introduced a ML-based approach for predicting DL and EE values in PLGA-based nanocarriers fabricated using microfluidic platforms. By mining a diverse literature-based dataset of over 25 variables, we applied feature selection and evaluated three regression models (SVR, GB, and RF). The RF model showed the highest predictive performance, with R^2^ values of approximately 0.96 for DL and 0.93 for EE, using only 10 optimized features. Additionally, we analyzed the potential interdependence between DL and EE and found minimal correlation, suggesting they can be optimized independently in formulation design. Local model explanations and cross-validation analysis further confirmed the robustness and interpretability of the selected models. As discussed in section “Comparison with DOE and advanced ML methods”, ML techniques—particularly ensemble models—offer a scalable, data-driven alternative to DOE-based optimization, capable of capturing complex nonlinear interactions with fewer experimental trials. Looking ahead, hybrid frameworks that integrate ML with DOE strategies or deep learning architectures may further improve prediction accuracy, interpretability, and experimental validation. Overall, our findings support the growing utility of ML in guiding the rational design of nanoparticle-based DDSs, offering a valuable complement—or even alternative—to traditional optimization methods in nanomedicine research.

## Supplementary Information

Below is the link to the electronic supplementary material.

Supplementary Material 1

## Data Availability

The data supporting the findings of this study were collected from published papers over the past decade. The data will be made available by the authors upon reasonable request. One of corresponding authors, Dr. Sima Rezavantalab, is responsible for sharing the data with interested parties.
