# Supplementary material for: Intelligence prediction of microfluidically prepared nanoparticles
Source: Sci Rep. 2025 Oct 27;15:37512. doi: 10.1038/s41598-025-21471-y (PMC12559373; doi:10.1038/s41598-025-21471-y)
Supplement: Supplementary file 1 — Supplementary Material 1 [file 41598_2025_21471_MOESM1_ESM.docx]

**Intelligence Prediction of Microfluidically Prepared Nanoparticles**

Nima Hanari^1^, Sara Mihandoost^1^, Sima Rezvantalab^2^

^1^ Electrical Engineering Department, Urmia University of Technology, Urmia 57166‑419, Iran;

^2^ Chemical Engineering Department, Urmia University of Technology, Urmia 57166‑419, Iran.

Corresponding author: [s.mihandoost@uut.ac.ir](mailto:s.mihandoost@uut.ac.ir) , [s.rezvantalab@uut.ac.ir](mailto:s.rezvantalab@uut.ac.ir)

| Table S1. List of therapeutic agents, chip types, and solvents that have been reported in papers. | | | | | |
| --- | --- | --- | --- | --- | --- |
| Drug | code | Chip type | code | Solvent | code |
| Curcumin | 1 | Glass | 1 | acetone | 1 |
| Bicalutamide | 2 | PEEK^[[1]](#footnote-1)^ | 2 | dichloromethane | 2 |
| Methyl jasmonate | 3 | PDMS^[[2]](#footnote-2)^- flow focusing | 3 | acetonitrile | 3 |
| Docetaxel | 4 | PDMS-3D | 4 | chloroform | 4 |
| Doxorubicin | 5 | PDMS-y | 5 | ethanol | 5 |
| Tamoxifen | 6 | PDMS-Multilaminar | 6 | methanol | 6 |
| Ribavirin | 7 | Glass-Herringbone | 7 | sodium oleate | 7 |
| SPION^[[3]](#footnote-3)^ | 8 | PDMS-Split & combine | 8 | DMSO^[[4]](#footnote-4)^ | 8 |
| Cisplatin | 9 | Plastic-Herringbone | 9 | THF^[[5]](#footnote-5)^ | 9 |
| Irinotecan | 10 | PDMS-Impact jet KM-3 | 10 | ethyl acetate | 10 |
| Ketamine | 11 | PDMS-baffle | 11 | HCL | 11 |
| p-DNA | 12 | P14 | 12 | DMF | 12 |
| PTX | 13 | Staggered herringbone | 13 | DMAB | 13 |
| Nifedipine | 14 | PDMS herringbone | 14 | TFE | 14 |
| BRP | 15 | PDMS-TrM | 15 |  |  |
| CQDs^[[6]](#footnote-6)^ | 16 |  |  |  |  |
| DNA | 17 |  |  |  |  |
| Ovalbumin | 18 |  |  |  |  |
| BSA^[[7]](#footnote-7)^ | 19 |  |  |  |  |
| Hybrid 56 | 20 |  |  |  |  |
| CK-10 | 21 |  |  |  |  |
| JO146 | 22 |  |  |  |  |
| Technetium-99m | 23 |  |  |  |  |
| Cotrimoxazole | 24 |  |  |  |  |
| Hydromorphone | 25 |  |  |  |  |

1. Polyether ether ketone [↑](#footnote-ref-1)
2. Polydimethylsiloxane [↑](#footnote-ref-2)
3. Superparamagnetic iron oxide nanoparticles  [↑](#footnote-ref-3)
4. Dimethyl sulfoxide [↑](#footnote-ref-4)
5. Tetrahydrofuran  [↑](#footnote-ref-5)
6. Carbon quantum dots [↑](#footnote-ref-6)
7. Bovine serum albumin [↑](#footnote-ref-7)
